# A phase 2, single-arm study of an autologous dendritic cell treatment against mucin 1 in patients with advanced epithelial ovarian cancer

**DOI:** 10.1186/2051-1426-2-16

**Published:** 2014-06-18

**Authors:** Paul LR Mitchell, Michael A Quinn, Peter T Grant, David G Allen, Thomas W Jobling, Shane C White, Anne Zhao, Vaios Karanikas, Hilary Vaughan, Geoffrey Pietersz, Ian FC McKenzie, Sharron E Gargosky, Bruce E Loveland

**Affiliations:** 1 Medical Oncology Unit, Austin Hospital, Olivia Newton-John Cancer and Wellness Centre, 145 Studley Road, Heidelberg, VIC 3084, Australia; 2 Royal Womens Hospital, 20 Flemington Road, Parkville, VIC 3052, Australia; 3 Mercy Hospital for Women, 163 Studley Road, Heidelberg, VIC 3084, Australia; 4 Monash Medical Centre, Clayton Road, Clayton, VIC 3168, Australia; 5 Burnet Institute, 85 Commercial Road, Melbourne, VIC 3004, Australia; 6 Monash University, Wellington Road, Clayton, VIC 3168, Australia; 7 Prima BioMed, Ltd., 151 Macquarie Street, Sydney, NSW 2001, Australia; 8 University of Melbourne, Parkville, VIC 3052, Australia; 9 Roche Innovation Center Zurich, 8952 Schlieren, Switzerland

**Keywords:** Ovarian cancer, Immunotherapy, CA125

## Abstract

**Background:**

Mucin 1 antigen, highly expressed by epithelial ovarian cancer (EOC), is a potential target for immunotherapy. A previous successful phase 1 trial was conducted in patients with adenocarcinoma who were injected with Cvac, autologous monocyte-derived dendritic cells (DCs) incubated with mannosylated mucin 1 protein (M-FP). The present study was a phase 2 trial of Cvac in patients with advanced EOC.

**Methods:**

Eligible patients had EOC with progressive disease, defined as an increase in CA125 of ≥ 25% in 1 month. The primary endpoint was CA125 response or stabilization. Peripheral blood mononuclear cells were collected by leukapheresis and cultured to generate DCs. The DC were incubated with M-FP, and after washing were prepared for injection into the patient intradermally every 4 weeks for 3 doses, then every 10 weeks for up to 12 months.

**Results:**

All 28 patients recruited were evaluable for safety and 26 for efficacy. All had undergone surgery and platinum-based chemotherapy, and 57% of patients received ≥ 3 chemotherapy regimens. There were no Grade 3 or 4 toxicities considered related to Cvac. Four patients showed CA125 response or stabilization (2 patients with major responses, 1 minor response, 1 stabilization) of median duration 10.3 months (5.3–16.3 months). An additional patient had > 25% CA125 reduction (not confirmed).

**Conclusions:**

Cvac immunotherapy was well tolerated. Clinical activity in EOC was evident based on decline or stabilization of CA125 in some patients, supporting ongoing development of Cvac in ovarian carcinoma and planning of additional trials of patients in remission is currently underway.

## Background

Epithelial ovarian cancer (EOC) causes approximately 125,000 deaths worldwide annually and in the United States is the fifth most common cause of cancer mortality in women [1]. The standard management of EOC for most patients involves surgical cytoreduction followed by platinum and taxane-based chemotherapy. While the majority of subjects achieve a clinical remission to this initial therapy, more than 70% relapse with incurable disease. Novel therapeutic approaches are needed to make substantial progress in this disease.

Mucin 1 is a glycoprotein that is highly expressed as a cancer-related protein variant in EOC and is thus a potential antigen for immunotherapy [2]. We and others have developed monocyte-derived dendritic cells (DCs) as vectors for cancer antigen delivery intended to enhance T-cell immune responses [3-7]. We have previously reported a phase 1 trial of Cvac, which involves the use of dendritic cells as a mechanism to induce a cellular response to mucin 1 [8]. We now report a phase 2 study of Cvac therapy in patients with EOC.

## Results

### Patients

Twenty-eight patients were recruited. Two patients discontinued for progression within 2 weeks. The characteristics of the other 26 patients (intent-to-treat [ITT] population) are detailed in Table 1. All patients were white but one. The median CA125 level at study entry was 758 U/mL (range 141–11,374 U/mL). As required per inclusion, CA125 levels ranged from 28-185% over the previous 1-month value, a CA125 median increase of 66% ( ≥ 25% was required for eligibility).

**Table 1:** Patient and disease characteristics of the ITT population (n = 26)

Age (years)	Median	58		
	Range	34–78		
			**Patients**	
ECOG performance status				
0			15	58%
1			10	38%
2			1	4%
Primary site:				
Ovarian			22	85%
Fallopian tube			1	4%
Peritoneum			3	11%
Histology				
Serous			22	85%
Endometrioid			1	4%
Endometrioid and clear cell			1	4%
Other			2	7%
Differentiation				
Grade 1			-	
Grade 2			1	4%
Grade 3			24	92%
Grade not assigned			1	4%
Tumor expression of mucin 1 by immunohistochemistry				
Positive			23	88%
Negative			2	8%
Not assessed			1	4%

All patients in the ITT population had previously received surgery and at least one course of platinum-based chemotherapy (Table 2). The median interval from completion of previous chemotherapy to first vaccination was 238 days (range 40–1412 days). Two patients had previous immunotherapy: one patient received autologous monocyte-derived activated killer (MAK) cells and the other autologous gamma delta T cells.

**Table 2:** Prior treatment of the ITT population (n = 26)

		**Patients**	
Surgery	26	100%
BSO	24	92%
TAH	22	85%
Omentectomy	25	96%
Chemotherapy			
Prior platinum regimen	26	100%	
1 prior line	9	35%	
2 prior lines	2	8%	
3 prior lines	7	27%	
4 prior lines	4	15%	
5 prior lines	4	15%	
Best response to most recent chemotherapy			
CR/NE	6	23%	
PR	5	19%	
SD	3	12%	
PD	11	42%	
Not known	1	4%	
Radiotherapy	4	15%	
Hormone therapy	3	12%	
Immunotherapy	2	8%	

BSO, bilateral salpingo-oophorectomy; CR, complete response; NE, not evaluable; PD, progressive disease; PR, partial response; SD, stable disease; TAH, total abdominal hysterectomy.

Five patients in the ITT population discontinued due to progression after 8–42 days. The remaining 21 patients comprised the per-protocol (PP) population.

### Treatment

Overall 34 leukaphereses were conducted in 26 patients, yielding a mean (±SEM) 2.5 ± 0.2 × 10^9^ peripheral blood mononuclear cells (PBMCs). After 6 days culture with granulocyte-macrophage colony-stimulating factor (GM-CSF) and interleukin 4 (IL-4), a mean of 19 ± 2 × 10^7^ viable cells (8% of the starting number) were recovered, and the first injection contained 4 × 10^7^ fresh cells. For subsequent injections, the Cvac had been frozen and was thawed prior to administration. The thawed Cvac cell product contained 1.5–5.7 × 10^7^ cells (mean of 3.7 ± 1.0 × 10^7^ and 89% ± 4% viability). Patients received a median of 3 vaccinations, and 2 patients received ≥ 7 vaccinations.

### Anti-tumor efficacy

**Figure 1:**
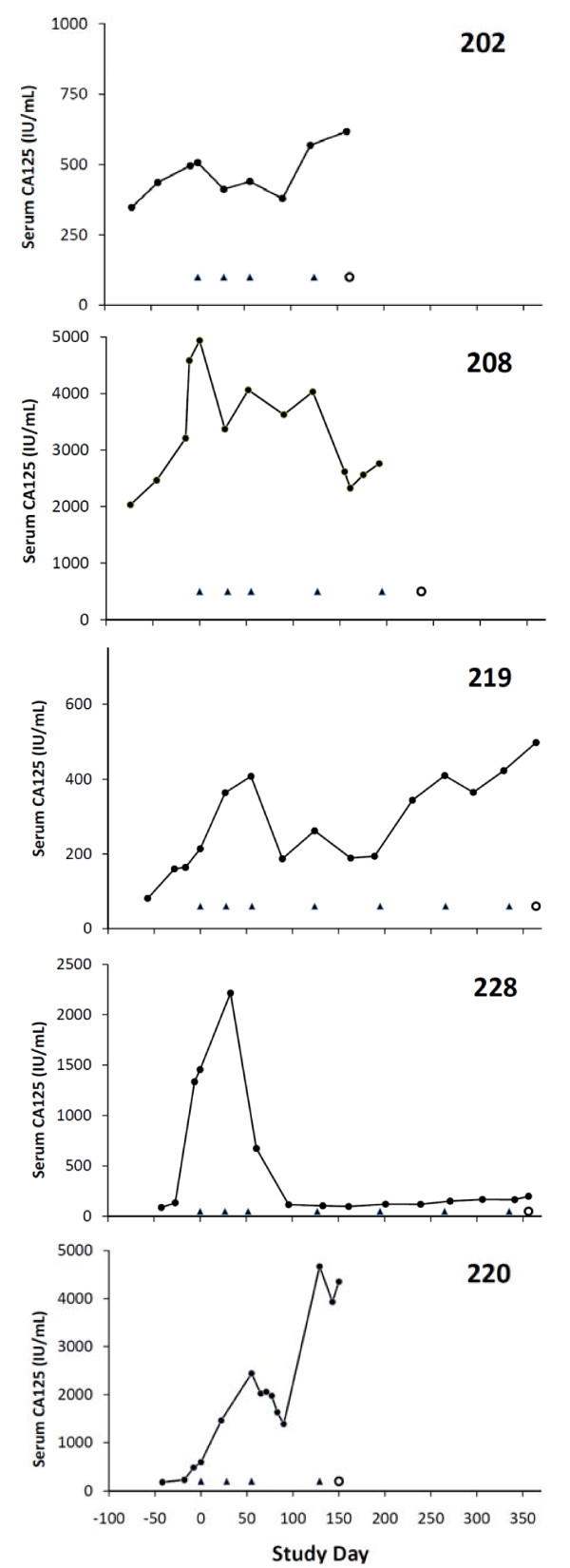
**Serum CA125 in the four confirmed (202, 208, 219, 228) and one unconfirmed (220) responding or stabilizing patients including the period of pretrial evaluation.** Cvac treatments began on day 1 and are indicated as (▲). The day of withdrawal from the trial is indicated as (**○**), except for patients 219 and 228 where data are truncated at 12 months.

The 26 patients evaluable for efficacy remained on study for a median 101.5 days (range 8–561 days). Four patients (15.4%) had response or stabilization according to CA125 criteria (patients 202, 208, 219, and 228) for 5.3–16.3 months (median 10.3 months). For the PP population defined as receiving three doses of treatment, the response/stabilization rate was 19% (4/21). A fifth patient (#220) had successive reduction in CA125 levels and a brief minor response in the weeks following the third treatment, which was not confirmed by a repeat CA125 1 month later (Table 3 and Figure 1). All 5 patients had serous histology with mucin 1-positive tumors, and there was no clear relationship to human leukocyte antigen (HLA) type (data not shown). For 3 of these patients, CA125 levels initially continued to increase 1.5–4.1-fold over the first 8 weeks after vaccination, before decreasing or stabilizing. For the 3 patients with confirmed major or minor responses, the nadir CA125 occurred around 5 months into treatment, while for the other 2 patients the nadir was around 3 months (Figure 1). These patients remained on study therapy for 5.3 to 18.5 months (Table 3). Patient 228, who had a major response and remained on study therapy for over 18 months, had previously received 3 chemotherapy regimens followed by immunotherapy with unmodified autologous gamma delta T cells (completed > 3 months before study entry, best response was progressive disease).

**Table 3:** Patients with CA125 response or stabilization

**Pt.**	**Age**	**Prior chemo regimens**	**Response latest treatment**	**Interval since latest treatment**	**CA125 at study entry (U/mL)**	**CA125 baseline for response (U/mL)**	**CA125 nadir (U/mL)**	**CA125 response (duration)**	**CT scan response**	**On study**
						**(study day)**	**(study day)**			
**Confirmed**										
202	76	4	PD	28 wks	507	507	379	Stable 23 wks	Target lesions stable at 24 weeks but new non-measurable lymphadenopathy	23 wks
						(d1)	(d91)			
208	58	1	PR	56 wks	4939	4939	2326	Minor 10 wks	Non-measurable disease only (ascites). New pleural effusion at 28 weeks	34 wks
						(d1)	(d161)	Stable 32 wks		
219	48	1	CR	54 wks	214	408	189	Major 10 wks	No abnormality on baseline or follow up scans	68 wks
						(d55)	(d163)	Stable 57 wks		
228	62	3 plus immuno-therapy	PD	16 wks	1455	2215	98	Major 71 wks	32% increase in measurable disease at 12 weeks, reduced to 24% at 28 weeks and 21% at 42 weeks	80 wks
						(d33)	(d161)			
**Unconfirmed**										
220	54	3	PR	20 wks	593	2442	1387	Minor 7 wks	At 14 weeks target lesions stable but new ascites	21 wks
						(d55)	(d90)	Stable 11 wks		

In general, disease seen on computed tomography (CT) scan was stable during CA125 stabilization or response (Table 3). One of the 2 patients with major response, both of whom were on study therapy for over a year, had a normal CT scan throughout (patient 219). The other major responder, Patient 228, had an initial increase in measurable disease (which would be progression by Response Evaluation Criteria in Solid Tumors [RECIST] criteria) then later a reduction in tumor size. It is noteworthy that this patient no longer needed regular palliative paracenteses after starting Cvac therapy.

All patients stopped Cvac therapy due to disease progression, with or without global clinical deterioration.

### Immunological response testing

#### Serum antibody response

Eight patients showed a weak mucin 1 antibody response (titers 1:40–1:80), which was pre-existing IgM in 5 patients (two of whom showed some seroconversion to IgG) and pre-existing IgG in 8 patients.

#### Cellular immunity (ELISPOT interferon gamma secretion results)

ELISpot assays performed on PBMC samples taken at the time of injections detected weak to moderate interferon gamma responses to positive control antigen or mitogen, but responses to mucin 1 were inconsistent and highly variable such that we were unable to detect any consistent or significant expansion of mucin 1-specific T cells in the circulation.

### Safety

#### Adverse events

No adverse events were considered definitely or probably related to study therapy.

Grade 2 (moderate) adverse events that occurred in more than one patient were gastrointestinal disorders in 9 patients (35%), fatigue in 5 patients (19%), and anemia in 2 patients (8%). There were 2 Grade 3 (severe) events (8%), abdominal pain and vomiting.

Three patients had lethargy that was considered possibly related and 2 patients had gastrointestinal events that were considered possibly related, one of whom also had influenza-like symptoms.

One serious adverse event not related was vomiting which led to withdrawal of therapy. This patient had progressive disease and died within a month of withdrawal.

#### Auto-antibody results

At baseline, anti-nuclear antibodies (ANA) were positive in 10 patients, anti-mitochondrial antibodies (AMA) and anti-thyroid antibodies were each positive in 2 patients, and 2 patients had mild elevation of rheumatoid factor. Twelve patients had follow-up testing at 4 months and 5 patients had follow-up testing at 12 months. Two patients became positive for ANA, 1 patient was positive for AMA at 4 months but negative at 12 months, and 1 patient who was positive for AMA at baseline became negative. There was no clinical evidence of autoimmune disease in these patients.

## Discussion

Mucin 1 antigen is highly expressed with aberrant glycosylation by many epithelial tumors and has been the focus of extensive cancer studies [2]. In murine studies carried out by our group, enhanced cellular immune responses were seen with conjugation of the recombinant mucin 1 antigen to oxidized mannan [9,10]. However, in our prior phase 1 trials when mannosylated mucin 1 was delivered by direct injection to over 100 patients with advanced mucin 1-positive malignancy, primarily antibody-mediated immunity was observed rather than cell-mediated immunity. No clinical responses were observed [11,12]. This may be due to naturally occurring human anti-Galα(1,3)Gal antibodies in cancer patients cross-reacting with mucin 1 peptides, leading to antigen clearance and antibody formation rather than cell-mediated immunity [13,14]. We then moved to a strategy of utilizing DCs for antigen delivery to promote a cell-mediated response, although patient benefit in studies carried out by others had been infrequent and inconsistent [15]. In our phase 1 trial in 10 patients with mucin 1-positive adenocarcinoma, Cvac was well tolerated, reliably led to T-cell immune responses, and resulted in stable disease in 2 patients, one with renal and the other with ovarian carcinoma [8].

The current trial was designed to determine whether Cvac might have activity in ovarian cancer. Responses to immunotherapy have been infrequent in other trials in progressive advanced EOC [16].

Immunotherapy is expected to be more effective in patients with minimal disease, and advanced metastatic cancer is a major hurdle. Thus efficacy of an immunotherapeutic agent in patients with advanced cancer would provide a strong rationale to develop the treatment. EOC has several characteristics that make it ideal for Cvac therapy: almost all cases are mucin 1 positive, advanced cases frequently are slowly progressive and often have good performance status, and CA125 is an easily performed and accepted surrogate marker of disease status and response to chemotherapy, although CA125 has not yet been validated as a surrogate for response to immunotherapy [1,17,18]. CT scans may be more difficult to interpret in patients with peritoneal disease and ascites, and 1 patient with renal carcinoma in the phase 1 study with Cvac had an initial increase in tumor size on CT scan, with subsequent tumor shrinkage and durable stable disease [8].

The current trial recruited patients with clearly progressive cancer based on CA125 criteria. We reasoned that the therapy might only lead to tumor stabilization, and in this situation the study would only provide clear results if all patients had been shown to have progressive disease at study entry. The minimum criterion of 25% increase in CA125 over 1 month (confirmed by a further CA125 result in all patients) was far exceeded by most patients, indicating rapidly progressive cancer despite a relatively long treatment-free interval for many patients.

Of 26 patients evaluable for efficacy, 4 patients had confirmed CA125 response or stabilization, which was durable, lasting 5–16 months. An additional patient had a minor response, which was brief and did not meet the CA125 criteria for confirmation. These patients all had clearly progressive cancer before starting Cvac, with 1.5–15.5-fold progressive increases in CA125 over 6–10 weeks prior to vaccination (Figure 1, Table 3). It is noteworthy that 3 of the 5 patients were heavily pretreated with 3 or more previous lines of chemotherapy, and that progressive disease was the best response to the most recent therapy for 11 patients.

The nadir CA125 indicating the best response to Cvac was not achieved until 3–5 months after starting Cvac therapy in these patients, and as also seen in our previous Cvac trial, there was an initial increase in CA125 in 3 patients for the first 1–2 months before response or stabilization became apparent. This has also been seen in other immunotherapy trials and supported our trial design, which allowed patients to stay on study for at least 3 months even if they met criteria for progression by CA125 or imaging criteria. A recent positive phase 3 study with ipilimumab immunotherapy in patients with melanoma allowed patients who progressed by RECIST criteria during the first 12 weeks to remain on treatment [19]. It is well recognized that patients may obtain long-term benefit from immunotherapy despite an initial increase in the size of tumor deposits, which may be due to therapy-induced intratumoral inflammation [20,21].

In addition, the delayed effect of immunotherapy may mean that patients have insufficient time to derive benefit from treatment that is interrupted by rapidly progressing disease and declining clinical status. The PP population, which excluded patients who discontinued after only 1–6 weeks and received only one or two vaccinations, achieved a response or stabilization rate of 4/21 (19%).

Imaging findings on CT scan generally paralleled the CA125 status. However, it is interesting to note that patient 228, who had a 70% decrease in CA125 at one month and 96% decrease at 4 months, had no reduction in disease on CT scan and instead had a 32% increase (progression by RECIST criteria) in disease from baseline at 4 weeks, which then later reduced to 21% above baseline at 42 weeks, but her clinical status reflected the decrease in CA125 as she was able to resume normal activities and was able to stop regular paracenteses after starting vaccination. This patient had previously received cellular immunotherapy resulting in disease progression, and although that therapy did not involve mucin 1 or any other antigen it is possible there was a priming effect for Cvac therapy.

Cvac therapy was well tolerated, and there was no evidence of clinical autoimmunity. Generally, patients were highly motivated, and cryopreservation of Cvac cell product allowed for infrequent leukapheresis.

In the current as well as the previous study [8], induction of anti-mucin 1 antibodies was infrequent. This was expected because of the delivery method whereby M-FP antigen was loaded into DCs *ex vivo* and was processed, allowing for the innate immune mechanism of DC presentation to T cells to occur. Circulating antigen was not available to induce an antibody response.

In the current study the ELISpot responses to positive control were weaker than in the previous study. We were unable to detect consistent and measurable levels above background of T-cell immune responses to the mucin 1 antigen, despite the observed clinical effects of the vaccine in the current trial and despite detecting clear T-cell responses in 9 of the 10 patients in our previous phase 1 trial of Cvac [8]. The low rate of T-cell reactions may indicate impaired immune response in the patients in the current trial, who had rapidly progressive disease at recruitment, or be due to cell handling or technical aspects, an issue we and others have experienced [22], especially as conduct of the assay had been transferred to a new laboratory. Furthermore, a low frequency of responding T cells in the peripheral circulation does not exclude the presence of disease-controlling effector T cells at disease sites.

Our results are especially important as there is no established immunotherapy for treatment of patients with EOC, and Cvac is well tolerated, unlike some immunotherapeutics. Although there has been considerable recent progress made in the immunotherapy of cancer [2,23], there have been only infrequent reports of responses in EOC [16,24,25]. Hernando et al. reported a Phase I study of patients with advanced gynecological malignancies vaccinated with DCs pulsed with keyhole limpet hemocyanin (KLH) and autologous tumor antigens derived from tumor lysate [26]. Three patients showed stable disease lasting 25–45 weeks, and 5 experienced early tumor progression within the first 14 weeks of beginning therapy. There have been a number of studies incorporating Her2-directed therapy. Chu *et al.*, in a Phase I/II trial of patients with advanced EOC in remission, evaluated vaccination with PBMC-derived DCs loaded with HER-2/neu, human telomerase reverse transcriptase and pan HLA-DR epitope peptides [25]. In this study, the peptide-loaded DC vaccination elicited only modest immune responses by ELISPOT assay, although overall survival of 90% at 3 years was very promising and there was no grade ≥3 toxicity. Brossart et al. studied patients with advanced breast and ovarian cancer vaccinated with autologous DCs pulsed with HER-2/neu- or MUC1-derived peptides [27], and was one of the earliest to show that vaccination with DCs pulsed with a single tumor antigen may induce immunologic responses in patients with ovarian cancer. Peethambaram et al. evaluated the use of lapuleucel-T (APC8024) in patients with HER-2/neu-expressing tumors [28]. Lapuleucel-T is an investigational active immunotherapy product consisting of autologous PBMCs, including APCs, which are cultured ex vivo with BA7072, a recombinant fusion antigen consisting of portions of the intracellular and extracellular regions of HER-2/neu linked to GM-CSF [28]. Therapy was well tolerated in the 18 patients with HER-2 expressing metastatic ovarian, colorectal and breast cancer and 2 patients experienced stable disease lasting >48 weeks.

Although CA125 has been widely used as a trial endpoint in EOC studies, this biomarker remains a surrogate for patient outcomes such as progression-free or overall survival, or a need to institute further anti-cancer therapy. While recognising CA125 as a surrogate endpoint, we have seen promising activity with Cvac in the current study based on CA125 response or stabilization in several patients.

In this, Cvac should not necessarily be seen as a competitor to chemotherapy or to current or future molecular targeting strategies such as bevacizumab, as Cvac may be complementary to such therapies. Recently there have been a number of reports of activity of CTLA4, PD1 and PDL1 inhibitors which block tumour-induced immune suppression, particularly in melanoma but also in other malignancies including ovarian carcinoma. The role of antigen-specific approaches such as CVac in combination with these inhibitors remains to be determined, but conceivably may be complementary. Additional studies will be required to determine this.

## Conclusions

The recent development of a closed production process for Cvac and cryopreservation of all cell product allows for a central facility for cell processing and delivery to remote treatment sites. The promising results of the current phase 2 study has led to further international trials with patients in remission or minimal residual disease, where Cvac therapy may have most benefit, allowing sufficient time for immunity to develop against a microscopic disease burden with hopefully greater therapeutic impact.

## Methods

### Study design

This was a single-center, phase 2 study conducted at the Austin Hospital in Melbourne, Australia. The vaccine consisted of each patient’s monocyte-derived DCs, harvested through leukapheresis of PBMCs, which were cultured *ex vivo* and incubated with recombinant mucin 1 fusion protein conjugated to mannan (M-FP), then injected back into the patient.

All patients were required to have clearly progressive disease at study entry. The primary objective of the study was stabilization or response as assessed by changes in CA125, which has been well validated as an endpoint [1,17,18].

The secondary endpoints were duration of response or stabilization, progression-free survival, safety, and immunological endpoints. Exploratory endpoints were relationship between response or stabilization and mucin 1 immunohistochemistry (IHC) status, histology, and HLA.

### Patients

Eligible patients had a pathological diagnosis of EOC, fallopian tube, or primary peritoneal carcinoma, with progressive disease at study entry based on an increasing CA125 level, defined as ≥ 25% increase in 1 month, confirmed by repeat CA125, with one level at least twice the upper limit of the normal range (ULN). Other eligibility criteria were: incurable disease; age ≥ 18 years; Eastern Cooperative Oncology Group (ECOG) performance status (PS) 0–2 (PS 2 patients were required to have no deterioration in PS and ≤ 10% weight loss in the previous 4 weeks); life expectancy ≥ 6 months; adequate hematologic (hemoglobin >10 g/dL, white blood cells > 3 × 10^9^/L, platelets > 100 × 10^9^/L), renal (creatinine < 160 mmol/L), and hepatic function (bilirubin < 2 × ULN, aspartate aminotransferase or alanine aminotransferase < 5 × ULN). Patients were excluded for: surgery, chemotherapy, radiotherapy, immunotherapy or experimental treatment within the previous 4 weeks; central nervous system metastases; ovarian sarcoma or mixed Müllerian tumor; another malignancy within 2 years except non-melanomatous skin cancer or non-invasive cervical cancer; active uncontrolled infection; any serious medical or psychiatric disorder compromising ability to give consent or comply with study procedures; concurrent systemic corticosteroid therapy; autoimmune disease other than autoimmune thyroid disease; clinically significant heart failure or ischemic cardiac disease; pregnancy or breast feeding. Patients were not required to have measurable disease nor to have tumors which were mucin 1 positive on IHC, given the high (>90%) frequency of mucin 1 expression in malignant ovarian cancer [29]. Nonetheless, tissue assessment of 27/28 showed mucin 1 positive staining.

Patients gave written informed consent and the study was approved by the Austin Health Human Research Ethics Committee.

Because 2 of the first 5 patients entering the study had rapid disease progression and were unlikely to derive benefit from an immunotherapy, the inclusion criteria were modified to exclude patients with PS 2 and with abnormal renal function.

### Response assessment

The primary endpoint was serum CA125 response. This was defined as a major response (≥50% reduction in CA125) or a minor response (≥25% reduction), confirmed by repeat CA125 after ≥ 3 weeks, or stable disease (<25% decrease and < 25% increase in CA125 for ≥ 3 months based on a minimum of 3 values). Progressive disease required CA125 increase by ≥ 25% above baseline, confirmed by a second measurement. The criterion for major response was consistent with the Gynecologic Cancer Intergroup criteria [17,18].

Cvac therapy was intended to induce an immunological response which would be expected to take some weeks to develop and months to be effective. In our previous phase 1 trial, a patient with EOC achieved prolonged stabilization of disease, but CA125 stabilization was not evident until 2 months from commencement of Cvac therapy [8]. Because a similar delay in the onset of antitumor effects was expected in the current study, patients were not considered to have progressive disease on CA125 criteria (or by changes on CT scans) during the first 3 months on study unless there was global clinical deterioration in the context of disease progression. This approach is consistent with that taken in the recently reported positive trial of ipilimumab immunotherapy in melanoma, where increasing disease size during the first 12 weeks was not considered evidence for lack of efficacy or reason to cease therapy [18].

For the same reason, in determining the CA125 response status, the baseline CA125 value was the greater of either the actual pre-vaccination level or the highest level during the first 3 months on treatment. It is important to note that the baseline level for assessing CA125 response was determined retrospectively and hence in some cases a corresponding CT scan was not performed at the same time point.

### Study procedures, investigations, and monitoring

Eligible patients underwent leukapheresis at baseline and as required to obtain additional PBMCs. After each leukapheresis, fresh Cvac cell product was used for the first vaccination, while additional cell product was cryopreserved and expected to provide sufficient cells for three or more further vaccinations.

CA125 was measured every 4 weeks until the third vaccination and then every 5 weeks. The autoantibody screen was measured at baseline, 4 and 12 months. Bloods for mucin 1 antibodies and ELISpot assay were taken at baseline and prior to each vaccination.

All patients had CT scans of the chest, abdomen, and pelvis at baseline. A CT scan was then repeated in patients on achieving a response or stabilization, and repeated every 3 months. The CT scan response assessment was according to RECIST 1.0 criteria [30].

### Cvac treatment

Patients received 3 cell product vaccinations over the first 8 weeks, then 4 more Cvac treatments at 10-weekly intervals for a total of 7 vaccinations in the first year. Patients with clinical benefit at 12 months may have continued vaccinations every 10 weeks. Cvac was administered by intradermal injection into 8 sites in the upper arms and thighs each of 5 × 10^6^ viable fresh cells (for the first treatment) in up to 0.2 mL per injection, and subsequently of thawed cryopreserved cells, aiming to inject a total of 25–40 million viable cells in up to 0.2 ml injections per site.

### Preparation of Cvac autologous vaccine

Cvac was prepared as previously described [8]. In brief, PBMCs were collected using a Haemonetics MCS plus machine, without priming, aiming over 2.5–3.5 hours to collect 3 × 10^9^ PBMCs. PBMCs were enriched under good manufacturing practice (GMP) conditions for DCs using standard monocyte adherence methods and cultured with rHu-GM-CSF (500 U/mL; Berlex, Richmond, CA) and rHu-IL-4 (200 U/mL; Gentaur, Brussels, Belgium). M-FP was manufactured under GMP conditions for the clinical trial [8]. M-FP (10 μg/mL) was added on culture day 5 for 18 hours (overnight), then non-adherent DCs were recovered.

The incubated cells were aliquoted for immediate patient administration or cryopreservation in vapor-phase liquid nitrogen for future doses as 10^7^ cells per 1 mL vial in autologous serum containing 11% glucose (Sigma, Saint Louis, MO) and 10% dimethyl sulfoxide (Sigma, Saint Louis, MO). We had previously demonstrated the phenotype and function of the DCs to be unaltered by cryopreservation (unpublished observations, Dr Loveland). The incubated DCs were CD86^+^, CD14^-^, and CD3^-^, with high expression of major histocompatibility complex (MHC) class I and II, CD83 low to moderate, CMRF44 moderate to strong, and the majority of cells positive for mannose receptor. Cell products were released for injection based on culture morphology (>50% DC-like), viability > 50%, and negative Gram stain. Cryopreserved cells were thawed, washed, and resuspended in 1.6 mL BP saline for injection (Baxter Corp, Sydney, Australia) with 2% v/v autologous serum. Leukapheresis cell product and Cvac cell product were monitored for phenotype by flow cytometry as previously described [8].

### Assessment of immune responses and mucin 1 immunohistochemistry

A standard ELISpot cytokine assay of PBMC T cells was carried out as previously described with the antigens PPD (positive control; Serum Statens Institut, Denmark) and recombinant pVNTR (specific mucin 1 test antigen) [8]. Anti-mucin 1 VNTR antibodies were measured by ELISA against pVNTR antigen [8].

MUC1 IHC using the BC2 monoclonal antibody on paraffin-embedded fixed tumor tissue was carried out as previously described [8]. Samples with > 50% of tumor cells strongly expressing mucin 1 were considered positive.

### Statistical considerations

As there was no standard immunotherapy in EOC, it was considered that evidence of clinical efficacy, whether stabilization or response, in even a single patient would be of interest. A sample size of 20 patients reaching at least 8 weeks after the first vaccination (minimum of 3 vaccinations) was selected considering the potential for patient recruitment. All patients recruited were included in the safety analysis, and patients with at least one post-baseline CA125 level were included in the ITT population and were evaluable for efficacy. The PP population included ITT patients who received at least 3 vaccinations and who remained on study for at least 8 weeks after the first vaccination.

## Abbreviations

AMA: Anti-mitochondrial antibodies; ANA: Anti-nuclear antibodies; EOC: Epithelial ovarian cancer; CT: Computed tomography; DC: Dendritic cell; ECOG: Eastern Cooperative Oncology Group; GM-CSF: Granulocyte-macrophage colony-stimulating factor; GMP: Good manufacturing practice; HLA: Human leukocyte antigen; IHC: Immunohistochemistry; IL-4: Interleukin 4; ITT: Intent-to-treat; MAK: Monocyte-derived activated killer; M-FP: Mannan-mucin 1 fusion protein; MHC: Major histocompatibility complex; PBMC: Peripheral blood mononuclear cell; PP: Per-protocol; PS: Performance status; RECIST: Response evaluation criteria in solid tumors; ULN: Upper limit of the normal range.

## Competing interests

The authors declare that they have no competing interests.

## Authors’ contributions

The following authors were clinical trial investigators and coordinators; PLRM, MAQ, PTG, DGA, TWJ, SCW. AZ, VK, HV, GP, IFCM and BEL were the manufacturing, scientific and clinical managers of the study. SG is the technical and scientific officer of the company sponsoring the trial. All authors read and approved the final manuscript.

## Authors’ information

Paul LR Mitchell conceived and designed the study with Ian F.C. McKenzie and Bruce E Loveland.
